# Information needs of parents of children with congenital anomalies across Europe: a EUROlinkCAT survey

**DOI:** 10.1186/s12887-022-03734-z

**Published:** 2022-11-12

**Authors:** Elena Marcus, Anna Latos-Bielenska, Anna Jamry-Dziurla, Ingeborg Barišić, Clara Cavero-Carbonell, Elly Den Hond, Ester Garne, Lucas Genard, Ana João Santos, LRenée Lutke, Carlos Matias Dias, Christina Neergaard Pedersen, Amanda J. Neville, Annika Niemann, Ljubica Odak, Anna Pierini, Juan Rico, Anke Rissmann, Judith Rankin, Joan K. Morris

**Affiliations:** 1 Population Health Research Institute, St George’s, University of London, Cranmer Terrace, London, SW17 0RE UK; 2 Chair and Department of Medical Genetics, Poznan University of Medical Sciences, Collegium Maius, Fredry 10, 61-701 Poznań, Poland; 3 Centre of Excellence for Reproductive and Regenerative Medicine, Children’s Hospital Zagreb, Medical School University of Zagreb, Ul. Vjekoslava Klaića 16, 10000 Zagreb, Croatia; 4 Rare Diseases Research Unit, Fundacio per al Foment de la Investigacio Sanitaria i Biomedica, Av. de Catalunya, 21, 46020 València, Spain; 5 Provincial Institute for Hygiene (PIH), Kronenburgstraat 45, 2000 Antwerp, Belgium; 6 Department of Paediatrics and Adolescent Medicine, Lillebaelt Hospital, University Hospital of Southern Denmark, Kolding, Denmark; 7 Department of Epidemiology, National Institute of Health Doctor Ricardo Jorge, Av. Padre Cruz, 1600-609 Lisbon, Portugal; 8 Department of Genetics, University Medical Center, University of Groningen, 9712 CP Groningen, Netherlands; 9 IMER Registry (Emilia Romagna Registry of Birth Defects), University of Ferrara and Azienda Ospedaliero Universitaria Di Ferrara, Via Aldo Moro, 8, 44124 Ferrara, Italy; 10 Medical Faculty, Malformation Monitoring Centre Saxony-Anhalt, Otto-Von-Guericke-University Magdeburg, Leipziger Str. 44, 39120 Magdeburg, Germany; 11 Unit of Epidemiology of Rare Diseases and Congenital Anomalies, Institute of Clinical Physiology, National Research Council, Via Giuseppe Moruzzi, 1, 56124 Pisa, Italy; 12 Population Health Sciences Institute, Newcastle University, Newcastle Upon Tyne, NE1 7RU UK

**Keywords:** Congenital anomaly, Child, Information needs, Support, Survey, Questionnaire

## Abstract

**Background:**

Parents of children who have a congenital anomaly can experience significant worry about their child’s health. Access to clear, helpful, and trustworthy information can provide a valuable source of support. In this study the aim was to explore the information needs of parents/carers of children with congenital anomalies across Europe.

**Method:**

A cross-sectional online survey was developed in nine languages to measure parents’ information needs, including: (1) the ‘helpfulness’/’trustworthiness’ of information received from eight relevant sources, and (2) overall satisfaction with information received. Parents/carers of children (0–10 years) with cleft lip, spina bifida, congenital heart defect [CHD] requiring surgery, and/or Down syndrome were recruited online via relevant organisations in 10 European countries from March-July 2021. Quantitative analyses using multivariable logistic regressions were performed.

**Results:**

One thousand seventy parents/carers of children with a cleft lip (*n* = 247), spina bifida (*n* = 118), CHD (*n* = 366), Down syndrome (*n* = 281), and Down syndrome with CHD (*n* = 58) were recruited in Poland (*n* = 476), the UK (*n* = 120), Germany (*n* = 97), the Netherlands/Belgium (*n* = 74), Croatia (*n* = 68), Italy (*n* = 59), other European countries (*n* = 92), and not specified/non-European countries (*n* = 84). Most participants were mothers (92%) and aged 31–40 years (71%). Participants were most likely to rate support groups (63%), patient organisations (60%), specialist doctors/nurses (58%), and social media (57%) as ‘very helpful’ information sources. ‘Very trustworthy’ ratings remained high for specialist doctors/nurses (61%), however, they declined for support groups (47%), patient organisations (48%), and social media (35%). Germany had the highest proportion of participants who were ‘very satisfied’ (44%, 95% CI = 34%-54%) with information, whereas this percentage was lowest in Croatia (11%, 95% CI = 3%-19%) and Poland (15%, 95% CI = 11%-18%). Parents of children with Down syndrome had significantly lower satisfaction ratings than parents of children with CHD; 13% (95% CI = 8%-18%) reported being ‘very satisfied’ compared to 28% (95% CI = 23%-33%) in the CHD group.

**Conclusions:**

Findings suggest that informal sources of information (e.g. support groups) are of value to parents, however, they are not deemed as trustworthy as specialist medical sources. Satisfaction ratings differed across countries and by anomaly, and were particularly low in Croatia and Poland, as well as for parents of children with Down syndrome, which warrants further investigation.

**Supplementary Information:**

The online version contains supplementary material available at 10.1186/s12887-022-03734-z.

## Background

Congenital anomalies (CAs) are structural or functional anomalies that are present from birth and are a leading cause of morbidity and mortality in children [1, 2]. The diagnosis of a CA is often an unexpected outcome for parents and can be very distressing [3–5]. Parents may feel anxious about their child’s health and prognosis [6, 7], and overwhelmed with information about an unfamiliar or complex medical diagnosis [5]. Many children with CAs will require ongoing support and treatment beyond the immediate postnatal period, and parents will need to learn how to manage their child’s healthcare needs [8, 9]. Ensuring parents have access to good quality information can reduce parental stress, help mitigate uncertainties, and empower parents to make decisions about their child’s care and wellbeing [9–12].

Qualitative research indicates that parents highly value receiving information from healthcare professionals (HCPs), as they are deemed to hold the position of experts, and thus be very trustworthy [8, 13, 14]. Parents value being reassured about their child’s CA [8, 13], and receiving condition-specific information (e.g. regarding symptoms/treatment) [8, 14], as well as information about access to services [14], the child’s development and the potential impact on the family [8, 13]. Personal interactions with HCPs have been found to have a positive impact on parental anxiety and to support coping [14, 15], especially when HCPs communicate information in a sensitive, empathic, and honest manner [13, 14, 16]. However, interactions with HCPs are not always adequate, and parents can leave appointments with questions unanswered [17–19], requiring them to seek information from alternative sources [14, 19–22].

Online health resources are easily accessible information sources which can offer helpful information for parents [23]. They are frequently used by parents to reduce anxiety [22] and to supplement information obtained from HCPs [24], however, they are not without issue. Parents have reported difficulties identifying relevant information [13], a propensity to find cases with poor outcomes [21, 25, 26], and challenges with interpretation [16]. The proliferation of social media (e.g. Facebook or blogs) in the mid-2000s has given patients and their families new ways to access health-related information and connect with peers for support [27]. Whilst research has suggested that these resources can empower patients [27], it is known that social media can promote inaccurate information [28], and therefore may lack reliability [29]. Parent organisations and charities which provide support and advice to affected individuals and their families may be able to bridge this “information gap” by sharing verified information. However, at present it remains unclear whether they are able to meet parents’ needs.

It is important to investigate parents’ and carers’ experiences with information, to understand whether their needs are being met and to inform how HCPs and government agencies may support them more effectively [14]. Using a cross-sectional online survey, this study aimed to explore parents’ and carers’ views about the information they have accessed, including satisfaction with information and whether further information was desired. The study was conducted as part of a collaborative European project, “Establishing a linked European Cohort of Children with CAs (EUROlinkCAT)” [30], which aims to investigate health and educational outcomes in children born with CAs using population-based data. This study investigated the information experiences of parents and carers of children with a cleft lip, spina bifida, a congenital heart defect (CHD), and/or Down syndrome in 10 European countries.

## Methods

This study is reported following the ‘Strengthening the Reporting of Observational studies in Epidemiology’ (STROBE) guidelines [31]. The survey was launched in the United Kingdom (UK) and Poland on 8 March 2021 and was available online until 14 July 2021 (and until 30 July in Italy). Ethics approval was granted by the St George’s University of London Research Ethics Committee on 18 December 2020 (reference number: 2020.0311), with further local ethics approvals obtained from each collaborating country (if required). The survey was launched in each country, as and when approvals were granted and translations were finalised (Table 1). The survey also explored parents’ and carers’ support needs (manuscript in preparation), and their experiences during the COVID-19 pandemic [32].

**Table 1:** Recruitment period and participant characteristics overall and by country group

Characteristic	All	UK	Poland	Germany	Croatia	Italy	Belgium/ Netherlands	Other EU^a^
**Recruitment period**								
Start date	-	8 Mar 2021	8 Mar 2021	11 May 2021	26 Apr 2021	16 Jun 2021	19 Apr 2021	6 Apr 2021
End date	-	14 Jul 2021	14 Jul 2021	14 Jul 2021	14 Jul 2021	31 Jul 2021	14 Jul 2021	14 Jul 2021
**N**	986	120	476	97	68	59	74	92
**Age**								
≤ 30	162 (17%)	18 (15%)	93 (20%)	13 (13%)	8 (12%)	4 (7%)	15 (20%)	11 (12%)
31–40	516 (53%)	53 (45%)	264 (56%)	51 (53%)	37 (55%)	27 (46%)	35 (47%)	49 (53%)
> 40	301 (31%)	47 (40%)	115 (24%)	33 (34%)	22 (33%)	28 (47%)	24 (32%)	34 (35%)
**Relation to child**								
Mother	911 (92%)	116 (97%)	449 (94%)	81 (84%)	63 (93%)	52 (88%)	64 (86%)	86 (95%)
Father	65 (7%)	2 (2%)	24 (5%)	13 (13%)	5 (7%)	6 (10%)	10 (14%)	5 (5%)
Other^b^	8 (1%)	1 (1%)	3 (1%)	3 (3%)	-	1 (2%)	-	-
**Employment**								
Employed	586 (60%)	81 (68%)	223 (47%)	61 (62%)	54 (79%)	44 (75%)	61 (82%)	62 (69%)
Homemaker/carer	301 (31%)	36 (30%)	198 (42%)	27 (29%)	7 (10%)	11 (19%)	8 (11%)	14 (16%)
Other^c^	94 (9%)	3 (3%)	52 (11%)	9 (9%)	7 (10%)	4 (7%)	5 (7%)	14 (16%)
**Education**								
School ≤ 18 years	390 (40%)	44 (37%)	163 (35%)	61 (67%)	19 (28%)	30 (52%)	44 (60%)	29 (32%)
University	482 (49%)	50 (42%)	257 (53%)	27 (29%)	45 (66%)	19 (33%)	29 (39%)	55 (60%)
Post-graduate	106 (11%)	25 (21%)	56 (11%)	3 (3%)	4 (6%)	9 (16%)	1 (1%)	8 (9%)
**Migrant status**								
> 10 years/from birth	924 (94%)	111 (93%)	467 (98%)	86 (88%)	64 (94%)	50 (86%)	71 (96%)	75 (81%)
6–10 years	30 (3%)	5 (4%)	5 (1%)	6 (7%)	2 (3%)	4 (7%)	1 (1%)	7 (8%)
1–5 years	28 (3%)	4 (3%)	2 (0.4%)	5 (5%	2 (3%)	4 (7%)	2 (3%)	9 (10%)
< 1 year	2 (0.2%)	-	1 (0.2%)	-	-	-	-	1 (1%)

Due to rounding, some sub-group percentages do not add up to 100%

^a^Other European countries: Denmark (*n* = 39), Portugal (*n* = 23), Spain (*n* = 16), Ireland (*n* = 5), Bulgaria (*n* = 2), Albania (*n* = 1), Cyprus (*n* = 1), Lithuania (*n* = 1), Norway (*n* = 1), Romania (*n* = 1), Sweden (*n* = 1), Ukraine (*n* = 1)

^b^Other family member (*n* = 3), legal guardian related to the child (*n* = 2), legal guardian unrelated to the child (*n* = 3)

^c^Unemployed (*n* = 56), long-term sick/disabled (*n* = 17), on furlough (*n* = 12), student (*n* = 8), retired (*n* = 1)

### Participants and recruitment

Participants were eligible if they (1) lived in Europe, (2) were parents, carers, or guardians (termed henceforth as *parents*) of a child up to 10 years of age, and (3) their child was diagnosed with a cleft lip, spina bifida, CHD which required surgery, and/or Down syndrome. These CA types were selected to cover a range of different impairments and varying familial experiences: (1) physical disability (spina bifida), (2) learning disability (Down syndrome), (3) visible anomaly (cleft lip), and (4) non-visible anomaly (CHD). Participants were recruited using convenience sampling in 10 European countries (Belgium, Croatia, Denmark, Germany, Italy, Netherlands, Poland, Portugal, Spain, UK). Relevant organisations in each country (see Supplementary file) advertised the survey online via their websites and social media, which included a link to the survey website. The study information sheet was available at the start of each language version of the survey. Participants either completed an online consent form, or consent was implied by submission of the survey (dependant on local ethics requirements). As the survey was shared across online platforms and by international organisations (e.g. the International Federation for Spina Bifida and Hydrocephalus), we received some responses from parents living in other countries. Responses from parents in other European countries (e.g. Ireland) were retained in the analysis, whereas those from non-European countries were excluded.

### Survey

The content of the survey was developed following a literature review of existing information needs questionnaires validated for patients or parents [33–44], and qualitative studies which explored the lived experience of parents of children with CAs [4, 6, 11, 13, 18, 23, 45–50], including a EUROlinkCAT qualitative study with parents of children with cleft lip, spina bifida, CHD and Down syndrome [16]. The conceptual framework was developed based on an existing information needs questionnaire validated for use in adults with cancer [35], which was adapted based on the qualitative studies to suit a CA population. The most notable changes were the inclusion of a question about ‘trust’ and the removal of questions about condition-specific needs. The survey was developed with input from expert clinicians, academics with expertise in questionnaire development and CA research. Six educators and parents provided feedback on the survey which resulted in changes to the survey length and the removal of technical terms. Time constraints of the project meant we were unable to conduct a full pilot of the final version of the survey.

The survey aimed to measure the extent to which parents’ information needs had been met by multiple information sources (helpfulness/trustworthiness), overall satisfaction, and whether there were any specific information gaps. The survey thus comprised the following sections (available in the supplementary file):

1. Parent demographics (7 items).
2. Child demographics/medical information (7 items).
3. Helpfulness of information (one item; rating eight information sources).
4. Trustworthiness of information (one item; rating eight information sources).
5. Satisfaction with information (one item).
6. Information topics (one item with 13 topics to choose from).

For Sects. (3) and (4), participants were asked to rate how helpful/trustworthy they found information received from a range of different sources: general practitioners (GPs), specialist doctors/specialist nurses, leaflets (from a HCP), research books/articles, patient/parent organisations, support groups/forums, social media, and internet searches (e.g. via Google). Outcomes across Sects. (3) to (5) were rated on 4-point Likert scales (e.g. “Not at all helpful-Very helpful” or “Not at all-Very much”). The terms “helpful” and “trustworthy” were not defined; this is because these terms tend to be judged differently by parents according to what is most important to their families’ needs, and we were interested in parents’ subjective perspectives as to what was helpful and trustworthy information (the extent to which their individual needs had been met). For Sect. (6) participants were asked to choose up to five topics they wanted more information about (out of 13 listed topics) or report that they did not require any further information. All items were close-ended and quantitative data were collected only. Items did not include a timeframe as the survey was aimed at all parents with a child up to the age of 10 and therefore specifying particular time points would have excluded participants with younger children.

The survey was developed in English and translated into eight European languages following existing guidance, including both a forwards and backwards translation [51]. The Dutch version was used both in Belgium and the Netherlands. Differences in education systems across countries meant that it was not possible to find equivalent terminology for parental education level. Categories were therefore chosen to represent local education systems in each country.

### Data collection

Survey data were collected using Research Electronic Data Capture (REDCap) tools [52] hosted at St George’s, University of London. All data collected were anonymous and no internet protocol (IP) addresses were collected, so preventing multiple participation was not possible. Participants were initially able to skip all survey items, however, an interim analysis (April 2021) revealed a large proportion of missing data for items about the parental country of residence and the child’s CA. These items were therefore made compulsory. We had a recruitment target of 80 participants per country which would have resulted in a standard error of 4.5% if 20% of participants replied to category 4 (very helpful to the question “how helpful did you find information accessed or received from your specialist doctor”), with 95% CI: 12%-30%. And if 40% of participants replied) to category 4 the standard error would be 5.5% with 95% CI: 29%-52%. Due to delays in obtaining ethics approvals, this target was not met for all countries.

### Data analysis

Data were downloaded from REDCap into Stata 17.0 software [53] and descriptive statistics were conducted. Respondents were asked to rate the ‘helpfulness’ and ‘trustworthiness’ of information from eight different sources, and the ratings were converted to a numerical score from 1 to 4 corresponding to from “very” to “not at all”. Principal components analysis (PCA) was performed on these eight ratings to create a smaller set of variables (components) that explained a large proportion of the variance in the dataset [54, 55], to aid the interpretation of potential patterns in the dataset. As is standard practice, only components that accounted for a significant proportion of the variance were selected (judged by an eigenvalue > 1) [56]. Each PCA (of the ‘helpfulness’ and ‘trustworthiness’ data) identified two principal components. A varimax orthogonal rotation was used to extract the components as it was assumed that the rating from different information sources were uncorrelated [57]. A scatterplot with axes corresponding to the two components were created to explore ‘helpfulness’ and ‘trustworthiness’ scores across the recruiting countries and the CA types.

All results (except the PCA findings) are presented only for countries with at least 50 participants. For the remaining countries, the data were combined into an ‘other European country’ group (termed *Other EU*), which comprised a heterogenous group of countries. Data for the Netherlands (*n* = 28) and Belgium (*n* = 46) were combined into a single group, due to similarities in survey response patterns (e.g. see Figs. 2 and 3), geographical location, and language. For CAs, data were categorised according to the four anomalies, and a fifth category created for children with Down syndrome and a CHD, as CHD is a common co-morbidity in children with Down syndrome [58]. It was not possible to create meaningful categories for children who had other combinations of the four anomalies, as there were too few participants (*n* = 15), and these were excluded from the regression analysis. Outcomes scored on 4-point Likert scales were dichotomised (very helpful/much vs. other responses) and modelled using multivariable logistic regressions which included the parental country of residence, age and education level, and the child’s CA type. The association of the parental country of residence and the anomaly type with outcomes was analysed using the contrast command in Stata 17.0. Age and education were included in the regression models as ordinal variables. To control for multiple comparisons, a significance level of 1% was adopted for all analyses.

It was not possible to calculate response rates because we used a multi-modal recruitment strategy, and we were unable to estimate how many parents were reached [59]. We report submission rates (i.e. the number of participants who started the survey/number who completed and submitted the survey) [60], and for those participants who submitted their survey, we calculated item-level response rates (the proportion of participants answering each item) for all outcomes [61].

## Results

### Participant characteristics

In total, 1,298 parents started the survey, of whom 1,109 (85%) submitted their responses. The submission rate varied from 78% in Italy to 92% in Belgium and Germany. An additional 123 (9.5%) submitted surveys were not included in the analysis because country of residence data were missing (*n* = 80), CA data were missing (*n* = 24), participants were from non-European countries (*n* = 4) or participants selected other combinations of the four CA types (*n* = 15). Item-level response rates were above 97% for all outcomes.

Of the 986 participants included in the analysis, the majority lived in Poland (*n* = 476). Other participants lived in the UK (*n* = 120), Germany (*n* = 97), Belgium/Netherlands (*n* = 74), Croatia (*n* = 68), Italy (*n* = 59), and the Other EU group (*n* = 92) which comprised participants residing in: Denmark (*n* = 39), Portugal (*n* = 23), Spain (*n* = 16), Ireland (*n* = 5), Bulgaria (*n* = 2), Albania (*n* = 1), Cyprus (*n* = 1), Lithuania (*n* = 1), Norway (*n* = 1), Romania (*n* = 1), Sweden (*n* = 1), Ukraine (*n* = 1). The majority of participants were mothers (92%), aged 31–40 years (71%), and employed (59%) (Table 1). In relation to education, 40% of participants had received technical training or formal education up to the age of 16 or 18, 49% had a university degree, and 11% had a post-graduate/doctoral degree. Only 60 participants (6%) reported that they had lived in their country of residence for less than 10 years.

### Child characteristics

Around a third of the sample were parents of children with CHD (*n* = 327; 33%). Other children were diagnosed with Down syndrome (*n* = 262; 26%), a cleft lip (*n* = 230; 23%), spina bifida (*n* = 112; 11%) and Down syndrome with a CHD (*n* = 55; 6%). A quarter of children had another CA, and 43% had another co-morbid health condition. The largest age category was 1–3 years (35%) and there was a slightly higher proportion of male children (56%). The majority of children were not yet of school age (62%), whereas 36% attended school and 2% were either home-schooled or unable to be schooled due to their health needs.

### Helpfulness and trustworthiness of the information sources

**Fig. 1:**
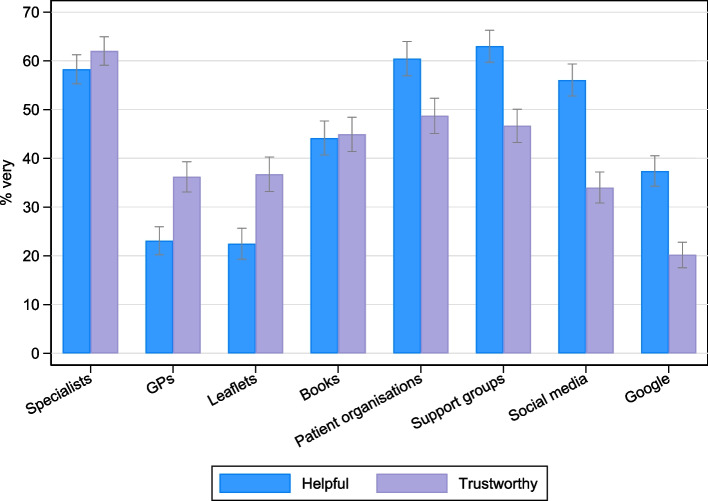
Proportion of participants rating each information source as ‘very helpful’ or ‘very trustworthy’, with 95% confidence intervals

Overall, the information sources with the highest proportion of ‘very helpful’ ratings were: support groups (63%), patient organisations (60%), specialist doctors/nurses (58%) and social media (57%) (Fig. 1). ‘Very trustworthy’ ratings were highest for specialist doctors/nurses (62%), followed by patient organisations (49%) and support groups (47%). Leaflets and GPs had the lowest proportion of ‘very helpful’ ratings, 22% and 24%, respectively. Internet searches had the lowest proportion of ‘very trustworthy’ ratings (20%). As shown in Fig. 1, there was a trend whereby medical sources of information (e.g. GPs) had higher ‘trustworthy’ than ‘helpful’ ratings, and non-medical sources of information (e.g. social media) had higher ‘helpful’ than ‘trustworthy’ ratings.

Participants with a higher level of education were significantly more likely to rate research articles/books as ‘very trustworthy’ sources of information (*p* < 0.001), and less likely to rate GPs as ‘very helpful’ (*p* = 0.008) or ‘very trustworthy’ (*p* = 0.003), or leaflets as ‘very helpful’ (*p* = 0.006). No age-related effects were found.

The analysis (PCA) of the ‘helpfulness’ data identified two principal components which explained 50% of the variance in the dataset. Table 2 shows the component loadings, which indicate how correlated the component is with the ratings for each individual data source. For the helpfulness data, the first component had relatively high positive loadings (i.e. > 0.4) for the non-medical data sources (such as support groups) and much smaller loadings for the medical data sources, indicating that the component is associated with the ratings from the non-medical sources and is not associated with ratings from medical sources; we therefore termed this component “informal sources”. Similarly, the second component had much higher positive loadings for the medical data sources with much smaller loadings for the non-medical sources, and therefore we termed this “medical sources”. The PCA of the trustworthy data resulted in the same two components, explaining 53% of the variance in the dataset (Table 2).

**Table 2:** Component loadings, eigenvalues, and the percentage of variance explained by each component, from the principal component analysis of the ‘helpfulness’ and ‘trustworthiness’ data

Variables	Helpfulness data		Trustworthiness data	
	**1 Informal sources**	**2 Medical sources**	**1 Informal sources**	**2 Medical sources**
General practitioner	-0.046	**0.573**	-0.064	**0.552**
Specialist doctor/nurse	-0.082	**0.616**	-0.098	**0.599**
Leaflets (from a HCP)	0.159	**0.515**	0.184	**0.526**
Research books/articles	**0.385**	0.072	**0.370**	0.179
Patient organisation	**0.435**	0.052	**0.430**	0.097
Support groups	**0.504**	-0.048	**0.497**	-0.057
Social media	**0.497**	-0.113	**0.487**	-0.159
Internet search	**0.358**	0.068	**0.382**	-0.007
Variance explained (%)	32.45	18.03	32.49	20.19
Eigenvalues†	2.596	1.442	2.599	1.615

*HCP* Healthcare professional

Component loadings > 0.300 are highlighted in bold. †Only components with eigenvalues > 1 are presented in this table

Helpfulness data – Kaiser–Meyer–Olkin Measure of Sampling Adequacy: 0.723; Bartlett’s Test of Sphericity: *p* < 0.001

Trustworthy data – Kaiser–Meyer–Olkin Measure of Sampling Adequacy: 0.679; Bartlett’s Test of Sphericity: *p* < 0.001

### Ratings across countries

**Fig. 2:**
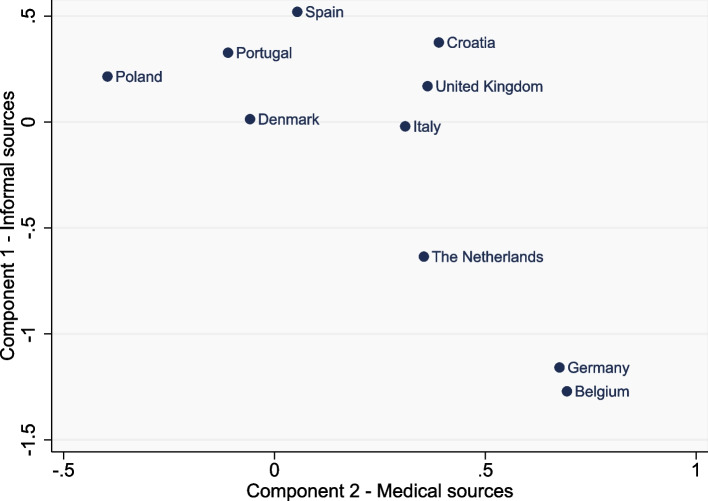
Mean scores for component 1 (informal sources) and component 2 (medical sources) of the ‘helpfulness’ data, by country

**Fig. 3:**
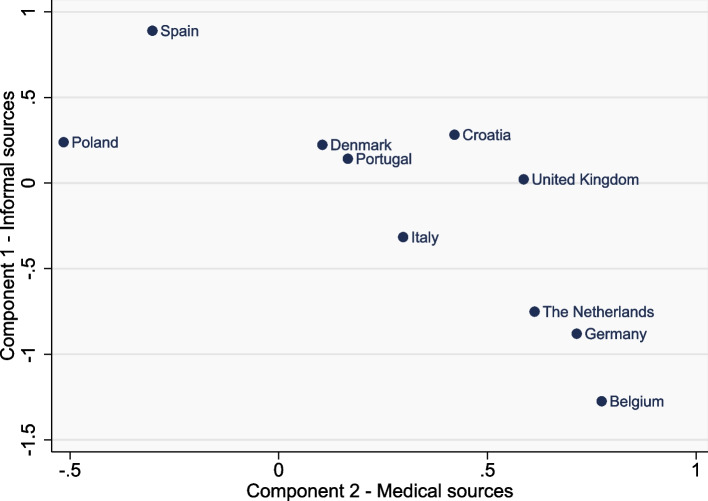
Mean scores for component 1 (informal sources) and component 2 (medical sources) of the ‘trustworthiness’ data, by country

Figure 2 and 3 illustrate the differences across countries in mean scores for each of the PCA components. For both the ‘helpfulness’ and ‘trustworthiness’ data, countries such as Germany, Belgium and the Netherlands tended to rate “medical sources” the highest amongst the countries, and rate “informal sources” lower than other countries. In contrast, countries such as Poland and Spain, tended to do the reverse with the lowest mean scores for “medical sources” and higher scores for “informal sources”. There was a significant impact of parental country on all information sources, except for internet searches. Supplementary Figure S1 and Figure S2 plot these results for specialist doctors/nurses and for support groups, for illustrative purposes. After controlling for age, education and CA type, Belgium/Netherlands had the highest percentage of ‘very helpful’ and ‘very trustworthy’ ratings for specialist doctors/nurses and the lowest percentage of ‘very helpful’ and ‘very trustworthy’ ratings for support groups. In contrast, Poland had a higher percentage of participants rating support groups as ‘very helpful’ or ‘very trustworthy’ compared to their ratings for specialist doctors/nurses (see Supplementary Tables S1-S2 for full ‘helpfulness’ and ‘trustworthiness’ ratings by country).

### Ratings across CA types

**Fig. 4:**
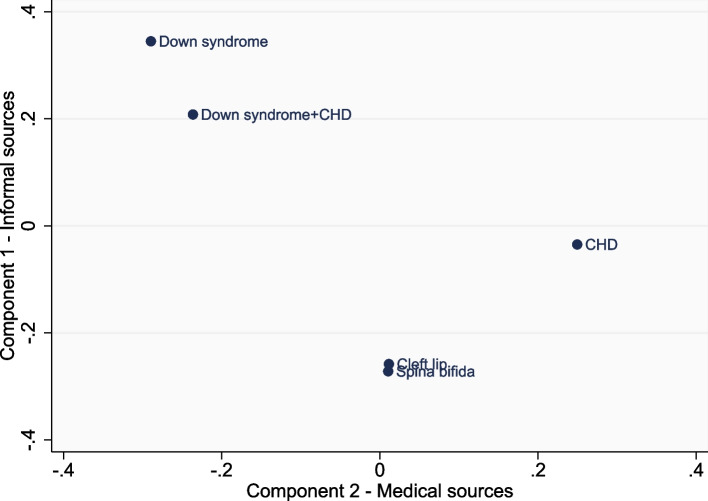
Mean scores for component 1 (informal sources) and component 2 (medical sources) of the ‘helpfulness’ data, by congenital anomaly type

**Fig. 5:**
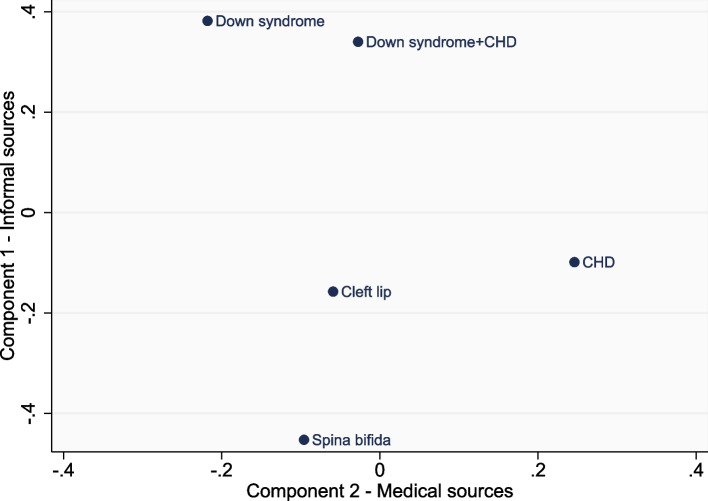
Mean scores for component 1 (informal sources) and component 2 (medical sources) of the ‘trustworthiness’ data, by congenital anomaly type

Figures 4 and 5 illustrate the differences across CA types in mean scores for each of the PCA components. For both the ‘helpfulness’ and ‘trustworthiness’ data, the cleft lip, spina bifida and CHD groups tended to score “informal sources” lower than the two Down syndrome groups. The CHD group had the highest scores for “medical sources”, for both the ‘helpfulness’ and ‘trustworthiness’ data. In contrast, the Down syndrome groups scored low for “medical sources”, although this was limited to the ‘helpfulness’ data. Another illustration of this is that after controlling for parental country, age, and education, parents of children with Down syndrome were significantly less likely to rate specialist doctors/nurses as ‘very helpful’ (39%, 95% CI: 32–45%) than parents of children with CHD (67%, 95% CI: 62–72%), and less likely to rate them as ‘very trustworthy’ (45%, 95% CI: 39–51%) compared with parents of children with CHD (69%, 95% CI: 64–74%). Parents of children with Down syndrome were also significantly less likely to rate leaflets (from a HCP) as ‘very helpful’ (16%, 95% CI: 11–22%) compared with parents of children with CHD (28%, 95% CI: 22–34%). No other CA effects were found across the other information sources and therefore full tables of these results are not provided.

### Satisfaction with information

Just under a quarter of participants (23%, 95% CI: 21–26%) across all countries were ‘very satisfied’ with the information they had received or accessed about their child’s health condition. This figure was lowest in Croatia (11%, 95% CI: 3–19%) and Poland (15%, 95% CI: 11–18%). Compared with Poland, satisfaction with information was significantly higher in Germany (44%, 95% CI: 34–54%; *p* < 0.001), Belgium/Netherlands (38%, 95% CI: 27–48%; *p* < 0.001), Italy (33%, 95% CI: 21–45%; *p* = 0.001), and the UK (32%, 95% CI: 22–39%; *p* < 0.001). In terms of CA type, parents of children with Down syndrome (13%, 95% CI: 9–18%) and Down syndrome with CHD (12%, 95% CI: 3–20%) had the lowest satisfaction ratings. For Down syndrome (alone), this was significantly lower than for parents of children with CHD (28%, 95% CI: 23–33%; *p* < 0.001). There was no impact of parental age or education level on satisfaction with information.

### Information topics

The topic that most parents wanted more information about was regarding their child’s ‘intellectual development’, with 51% of parents picking this option (see Supplementary Table S3 file for full ratings). This was followed by ‘treatment options’ (43%), and ‘physical development’ (40%). Approximately a third of parents wanted more information about ‘support with school/education’ (35%), ‘positive information about my child’s full potential’ (34%), ‘diet and feeding’ (33%), and ‘specialist medical centres’ (32%).

When controlling for parental country, age, and education level, there was a significant impact of CA type on each of the three most commonly picked topics. For ‘intellectual development’, parents of children with Down syndrome (73%, 95% CI: 67–78%; *p* < 0.001), and Down syndrome with CHD (72%, 95% CI: 60–84%; *p* = 0.003), were significantly more likely to pick this as a topic of interest, compared with parents of children with CHD (49%, 95% CI: 44–55%). Parents of children with spina bifida had the highest ratings for ‘treatment options’ (62%, 95% CI: 54–71%), and were significantly more likely to pick this topic compared to parents of children with CHD (47%, 95% CI: 42–52%; *p* = 0.005), whereas parents of children with Down syndrome were significantly less likely to (*p* < 0.001). For ‘physical development’, the highest ratings came from parents of children with spina bifida (53%, 95% CI: 44–62%) and CHD (51%, 95% CI: 45–56%), and this latter group (CHD) were significantly more likely to pick this information topic compared with parents of children with a cleft lip (23%, 95% CI: 17–29%; *p* < 0.001).

Other highly rated topics for parents of children with Down syndrome were ‘support with school/education’ (52%), ‘diet and feeding’ (50%), and ‘positive information about child’s full potential’ (46%) (Supplementary Table S3 file). For parents of children with spina bifida and cleft lip, the other highly rated topic was ‘specialist medical centres’, with 46% and 33% of parents indicating they wanted more information of these, respectively.

## Discussion

### Main findings

To our knowledge this is the first study to explore the information needs of parents of children with different CAs across several European countries. Overall, only around a quarter of participants reported being very satisfied with the information they had accessed, suggesting a high level of unmet need, which was particularly apparent in Poland and Croatia. There was a trend for informal sources of information (e.g. social media) to have higher ‘helpfulness’ than ‘trustworthiness’ ratings, and vice-versa for medical sources of information (e.g. GPs). Support groups, patient organisations, specialist doctors/nurses and social media were deemed the most helpful sources of information by parents, however, whereas trustworthy ratings remained high for specialist doctors/nurses, they declined for these other informal sources. GPs and leaflets were rated as the least helpful information sources, whereas internet searches and social media had the lowest trustworthy ratings. These findings support previous research indicating a high level of trust in medical specialists [13, 20], the importance of engaging in dialogue with professionals [4, 14, 18] as opposed to receiving passive information, and a lack of knowledge about CAs from general healthcare staff [6, 49]. Connecting with peers has also been deemed useful by parents [24, 62, 63], which was reflected in our survey, with both support groups and patient organisations rated very highly for ‘helpfulness’. Lower ‘trustworthiness’ ratings for these sources may in part be explained by conflicting information parents can receive within support groups [14]. Interestingly, despite the fact that there are fewer non-English resources available online, we found no significant differences across countries in helpfulness/trustworthiness ratings for the internet, which in keeping with previous research [13, 16, 20, 26], was consistently rated poorly.

There were regional differences in ‘helpfulness’ and ‘trustworthiness’ ratings, with Poland and Spain scoring lower for medical sources of information compared with other countries, and higher for informal sources. In contrast, Belgium, Netherlands and Germany tended to score highly for medical sources and lower for informal sources. Although cultural differences may partly underpin these differences, these findings may also reflect the capacity of healthcare systems within each country to meet the needs of parents. One potential explanation could be that more highly resourced centres are able to provide more contact with medical specialists or deliver more personalised information, with HCP staff shortages resulting in a more acute parental need for information [20]. Figures from the Organisation for Economic Co-operation and Development (OECD) in 2018 indicate that Poland had one of the lowest numbers of practising nurses per head in Europe (5.1/1,000 population), whereas Germany, Belgium and the Netherlands had some of the highest (11.1–13.2/1,000) [64]. When information needs are not met by professionals, patients tend to conduct their own searches [14, 16, 20]. It is possible that parents in countries with staff shortages may have had a greater need to develop informal resources such as support groups and parent organisations.

Parents of children with Down syndrome had the lowest ratings for overall satisfaction with information and they also scored medical sources of information as less helpful and trustworthy than parents of children with other anomalies. This may in part be due to the fact that specialist doctors are able to intervene in conditions such as CHD, whereas for intellectual disabilities associated with Down syndrome, there are limitations to what specialist doctors can address. There may also be a certain level of social stigma still associated with Down syndrome, with professionals feeling that these children may not be accepted in society [65]; this in turn may affect their interactions with parents. In Buyukavci et al. (2019), mothers of children with Down syndrome described being dissatisfied with professionals’ attitudes, and reported that they focused discussions on the child’s limitations and negative health outcomes, rather than potential achievements [66]. In our survey, around a third of participants overall reported wanting more information about ‘positive information about my child’s full potential’, although interestingly this figure was notably higher among parents of children with Down syndrome (46%), suggesting this was a higher priority topic for this group.

### Strengths and limitations

Overall, a large sample of parents were recruited, and the proportion of each CA type also reflects the relative number of live births with each CA in Europe [67]. Including parents of children with different CA types within several countries, allowed a broad range of experiences to be collected. The use of an online convenience sampling strategy meant we were unable to calculate response rates and there is a risk of selection bias; our findings may not be generalisable to all parents of children with CAs, especially people living with ‘digital poverty’ who are unlikely to have taken part. The majority of participants lived in Poland, which for the overall study estimates, limits the generalisability of findings to parents across the rest of Europe. The most successful recruitment strategy was via patient and parent organisations, and therefore we may have excluded parents who do not engage with these organisations, whose experiences may differ from our sample. A consistent recruitment strategy was used across countries, however, each country differed in the number of organisations who were able to support recruitment, and the frequency with which they were able to post study adverts. It is possible that these variations may underpin some of the differences found across countries. We were mindful to avoid leading questions and complex/ambiguous language when developing the survey, however, we were unable to formally test this.

### Implications and future research

This survey found that online information resources, whilst helpful, were rated relatively low for ‘trustworthiness’. With trust in these information sources lacking, it is important that HCPs actively signpost parents to reliable sources of information, such as clear medical websites. Patient and parent organisations may benefit from spending more time developing the information they share, ensuring it is evidence-based, up-to-date and reliable. Involving academics and clinicians in the generation of this information would support the development of resources that are both trustworthy and accessible to parents. There are limitations to how much time HCPs can spend with parents, and as such, new strategies to improve information from these organisations could help fill this information gap. Non-specialist medical sources of information (GPs and leaflets) were rated very low for ‘helpfulness’. This is likely due to the fact that GPs are specialised in common medical conditions, and may only meet few children with a CA during their career; one would therefore not expect them to have specialist knowledge about these rarer conditions. As the purpose of leaflets is to be relevant to all families, they need to be kept general, and therefore they may lack information which meets the specific and diverse needs of each child and their family. Of note is the fact that parents sometimes request information that is not available, and therefore medical specialists may always have a gap in knowledge. For example, school performance is a research topic of interest to parents, however, it has a very limited evidence base for these CA types [68]. Despite these facts, medical professionals may also benefit from exploring informal sources of health information, such as social media and patient organisations, to understand exactly what kind of information parents find helpful, and to seek reliable sources which they can signpost parents to. Further research on the information needs of parents at different time points will be important to explore. Most of the available literature has focused on parents’ needs around diagnosis, and it would be useful to explore how their needs might differ in later years, to ensure information from organisations and professionals is targeted. It will also be of interest to explore why information from patient and parent organisations may be perceived as less trustworthy than HCPs, and whether there may be specific solutions to improve the reliability of this information.

## Conclusion

Our findings indicate that parents obtain information about their child’s health condition from a range of medical and informal sources. Informal sources were found to be highly valued by parents, however, medical specialists had the highest ratings for both ‘helpfulness’ and ‘trustworthiness’. In contrast GPs and leaflets (from a HCP) were rated very low for ‘helpfulness’. Overall satisfaction with information was somewhat low and indicates a potential information gap, especially in Croatia and Poland, and for parents of children with Down syndrome.

## Supplementary Information

**Additional file 1.**

## Data Availability

The datasets analysed during the current study are available from the corresponding author on reasonable request.
