# Supplementary material for: Information needs of parents of children with congenital anomalies across Europe: a EUROlinkCAT survey
Source: BMC Pediatr. 2022 Nov 12;22:657. doi: 10.1186/s12887-022-03734-z (PMC9652126; doi:10.1186/s12887-022-03734-z)
Supplement: Supplementary file 1 — Additional file 1. [file 12887_2022_3734_MOESM1_ESM.pdf]

SUPPLEMENTARY FILE

(A) TABLES AND FIGURES

Table S1 Proportion* of participants reporting that information accessed/received from each of the following sources was 'very helpful', by country.

Country	Specialist doctors/nurses (n†=918)	GPs (n=†767)	Leaflets (n=†638)	Research books/articles (n=†756)	Patient organisations (n=†727)	Support groups (n=†747)	Social media (n†=816)	Internet searches (n†=919)
	**%** [95% CI]	**%** [95% CI]	**%** [95% CI]	**%** [95% CI]	**%** [95% CI]	**%** [95% CI]	**%** [95% CI]	**%** [95% CI]
Poland	46 [41, 51]	18 [14, 22]	18 [14, 23]	50 [45, 55]	65 [59, 70]	73 [68, 78]	68 [64, 73]	41 [37, 46]
UK	68 [60, 76]	18 [10, 26]	27 [18, 35]	31 [22, 39][1]	54 [44, 64]	55 [46, 65]	49 [39, 58]	32 [23, 40]
Croatia	65 [54, 76]	37 [24, 49]	29 [15, 43]	43 [30, 56]	53 [39, 66]	64 [51, 76]	53 [40, 65]	34 [21, 45]
Italy	74 [62, 86]	36 [23, 49]	10 [2, 19]	36 [23, 49]	82 [72, 93]	48 [33, 63	44 [29, 58	30 [18, 43]
Germany	75 [66, 84	37 [26, 47]	38 [25, 51]	67 [51, 82]	60 [47, 74]	61 [48, 74]	47 [33, 61]	39 [29, 50]
Belgium/Netherlands	77 [67, 87]	20 [10, 30]	17 [8, 26]	27 [14, 39]	33 [19, 47]	23 [10, 35]	21 [10, 32]	21 [11, 30]
Other EU	59 [49, 69]	19 [10, 28]	29 [18, 39]	43 [31, 54]	58 [47, 69]	60 [49, 71]	44 [34, 55]	42 [32, 52]
Total	58 [55, 61]	24 [21, 27]	22 [19, 26]	44 [41, 48]	60 [57, 64]	63 [59, 66]	56 [53, 60]	38 [35, 41]
Heterogeneity between countries	p<0.0001	p=0.0003	p=0.0064	p=0.0003	p=0.0001	p<0.0001	p<0.0001	p=0.0278

*Adjusted by congenital anomaly type, parental age, and education level. Unadjusted proportions are not included in this table.
†Total number of participants completing the item, excluding 'not applicable' responses. Missing data: specialist doctor/nurse (n=10), GP (n=9), leaflets (n=15), research books/articles (n=18), patient organisations (n=24), support groups (n=18), social media (n=17), internet searches (n=13).
CI = confidence interval; GP = general practitioner

Table S2 Proportion* of participants reporting that they found the information received or accessed from the following sources 'very trustworthy', by country.

Country	Specialist doctors/nurses (n†=911)	GPs (n†=740)	Leaflets (n†=639)	Research books/articles (n†=740)	Patient organisations (n†=725)	Support groups (n†=761)	Social media (n†=811)	Internet searches (n†=888)
	**%** [95% CI]	**%** [95% CI]	**%** [95% CI]	**%** [95% CI]	**%** [95% CI]	**%** [95% CI]	**%** [95% CI]	**%** [95% CI]
Poland	46 [41, 50]	20 [15, 24]	22 [17, 27]	45 [40, 50]	51 [46, 57]	55 [50, 60]	42 [37, 47]	23 [19, 27]
UK	75 [67, 82]	33 [23, 43	47 [37, 56]	30 [21, 39]	35 [26, 45]	35 [26, 44]	20 [12, 27]	15 [8, 21]
Croatia	79 [69, 88]	45 [32, 58]	38 [22, 54]	59 [46, 72]	46 [32, 59]	36 [23, 48]	25 [14, 36]	18 [9, 28]
Italy	73 [60, 85]	62 [48, 77]	28 [14, 42]	26 [14, 40]	72 [59, 85]	35 [21, 50]	34 [19, 48]	20 [8, 31]
Germany	83 [75, 91]	70 [60, 81]	73 [61, 84]	70 [56, 84]	56 [42, 70]	55 [42, 68]	42 [29, 55]	22 [13, 31]
Belgium/Netherlands	80 [70, 90]	61 [47, 75]	45 [32, 59]	46 [31, 60]	26 [12, 39]	19 [8, 31]	13 [4, 21]	8 [2, 14]
Other EU	71 [61, 80]	35 [23, 46]	47 [34, 59]	51 [40, 63]	50 [39, 61]	45 [34, 57]	28 [18, 37]	25 [15, 34]
Total	62 [59, 65]	37 [33, 41]	37 [33, 41]	45 [41, 49]	49 [45, 52]	47 [43, 50]	34 [31, 37]	20 [17, 23]
Heterogeneity between countries	p<0.0001	p<0.0001	p<0.0001	p<0.0001	p=0.0001	p<0.0001	p<0.0001	p=0.0813

*Adjusted by congenital anomaly type, parental age, and education level. Unadjusted proportions are not included in this table.

†Total number of participants completing the item, excluding 'not applicable' responses. Missing data: specialist doctor/nurse (n=13), GP (n=11), leaflets (n=21), research books/articles (n=19), patient organisations (n=21), support groups (n=15), social media (n=35), internet searches (n=14).

CI = confidence interval; GP = general practitioner

Table S3 Percentage of participants reporting that they would like more information about each topic, in the total sample and by each congenital anomaly group

**Topic**	**Total** n=986	**Cleft Lip** n=230	**Spina bifida** n=112	**Congenital heart defect** n=327	**Down syndrome** n=262	**DS + CHD** n=55
Intellectual development	**51%**	**30%**	37%	**48%**	**74%**	**75%**
Treatment options	**42%**	**43%**	**64%**	**43%**	30%	40%
Physical development	**40%**	22%	**52%**	**50%**	40%	33%
Support from school	35%	27%	39%	24%	**52%**	**48%**
Positive information about child’s potential	34%	21%	34%	34%	46%	38%
Diet and feeding	33%	22%	20%	31%	**50%**	**44%**
Specialist medical centres	32%	**33%**	**46%**	27%	33%	25%
Quality of life	30%	14%	33%	43%	28%	27%
Financial support	24%	28%	34%	16%	27%	29%
Exercise	19%	16%	28%	23%	15%	13%
How to meet other parents	17%	23%	18%	17%	13%	11%
Sleep	11%	7%	3%	9%	18%	18%
Patient organisations/support groups	11%	15%	10%	12%	8%	15%
No more information required	8%	17%	4%	7%	3%	4%

Percentages highlighted in **bold** represent the highest three scoring topics for each column.
DS – Down syndrome; CHD – congenital heart defect.

Figure S1 Proportion* of participants rating each information source as 'very helpful', with 95% confidence intervals, by country.

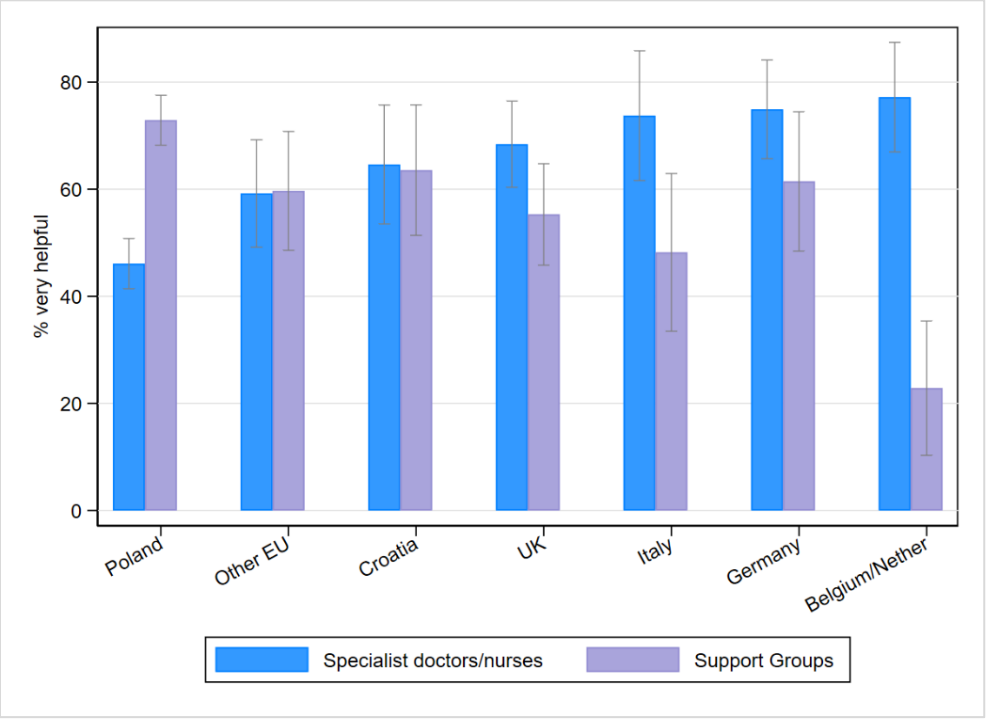

*Adjusted by congenital anomaly type, parental age, and education level.

Figure S2 Proportion* of participants rating each information source as 'very trustworthy' with 95% confidence intervals, by country.

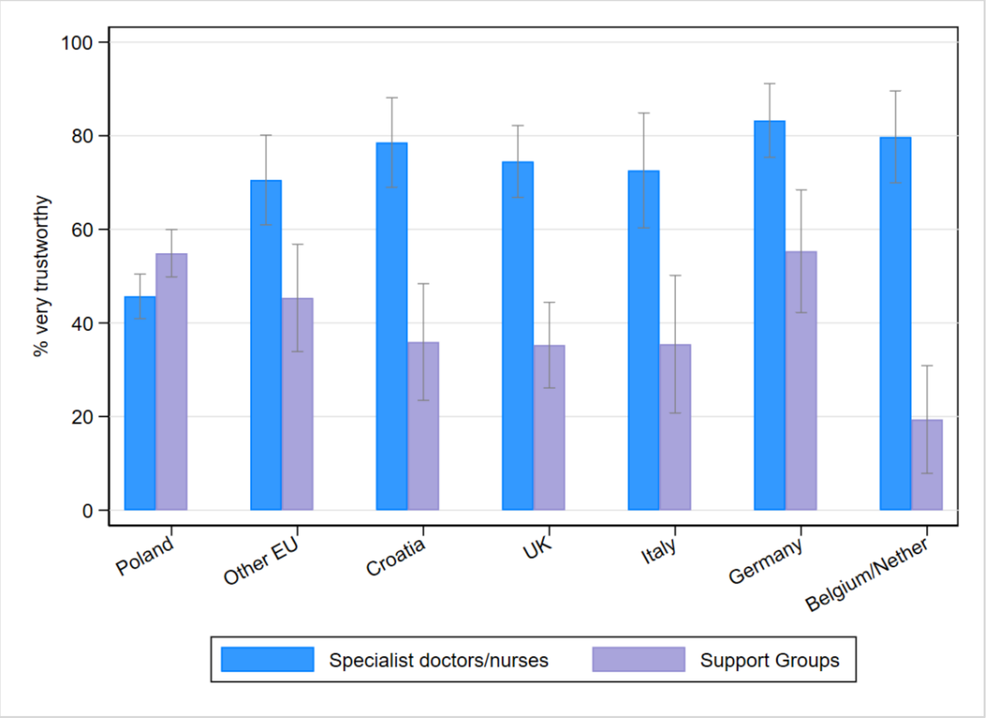

*Adjusted by congenital anomaly type, parental age, and education level.

(B) SURVEY ITEMS

(1) Parent Demographics

1. Which country do you live in? [drop-down list]

2. What type of area do you live in?

a. City (population over 500,000)
b. Large town (population between 100,000 and 500,000)
c. Medium town (population between 20,000 and 100,000)
d. Small town (population less than 20,000)
e. Suburban village
f. Village
g. Rural/isolated area (e.g. a farm)

3. What is your age?

a. Less than 20 years
b. 20-25 years
c. 26-30 years
d. 31-35 years
e. 36-40 years
f. 41-45 years
g. 46-50 years
h. More than 50 years

4. What is the highest level of education you have completed?

a. Primary school
b. Secondary school up to 16 years
c. Secondary or further education after 16 years
d. University
e. Post-graduate / Doctoral studies

5. What is your employment status?

a. Employed (full-time), including self-employed
b. Employed (part-time), including self-employed
c. Full-time homemaker/carer
d. Long-term sick/disabled
e. Retired
f. Student
g. Unemployed
h. On furlough

6. How long have you lived in your country of residence?

a. Up to 1 year
b. Between 1-5 years
c. Between 6-10 years
d. More than 10 years
e. From birth
f. Prefer not to say

7. What is your relationship to the child this survey is about?

a. Mother (biological)
b. Mother (adoptive)
c. Father (biological)
d. Father (adoptive)
e. Legal guardian related to the child
f. Legal guardian unrelated to the child / foster parent
g. Another family member

(2) Child Demographics and Medical Information

1. What age is your child?

a. Less than 1 year
b. 1-3 years
c. 4-6 years
d. 7-10 years

2. What is your child's gender?

a. Male
b. Female
c. Other
d. Prefer not to say

3. Which of the following conditions has your child been diagnosed with? (If your child has more than one of these conditions, please select all that apply)

a. Cleft lip (with or without cleft palate)
b. Spina bifida
c. Congenital heart defect that required surgical intervention
d. Down's syndrome

4. Was your child's [*condition*] detected prenatally (during pregnancy)?

a. Yes [survey moves to question 5]
b. No [survey skips to question 6]
c. I don't know [survey skips to question 6]

5. In which week of pregnancy was your child's [*condition*] detected?

a. Before 13 weeks
b. Between 14 and 21 weeks
c. At 22 weeks or later
d. I'm not sure

6. Does your child have any other congenital anomalies (conditions present from birth)?

a. Yes

Please select all that apply:

- Brain anomalies
- Hydrocephalus
- Eye anomalies
- Anomalies of face, ear and neck

- Lung anomalies
- Abdominal anomalies
- Renal anomalies
- Genital anomalies
- Skeletal anomalies
- Limb anomalies
- Chromosomal or genetic abnormality (other than Down's syndrome)
- Other anomaly

b. No

7. Does your child have any other health conditions?

a. Yes

Please select all that apply:

- Autism or attention disorder
- Learning disability
- Epilepsy
- Cerebral Palsy
- Asthma
- Allergy or food intolerance
- Eczema or other skin disease
- Recurrent infections
- Hearing loss
- Vision problems
- Celiac disease
- Diabetes
- Endocrine disorder
- Immune disorder
- Blood disorder
- Cancer
- Other

b. No

(3) Helpfulness of information

1. Please rate how helpful you have found information accessed or received from the following sources about your child's condition. (If you have not accessed or received information from a listed source, please select N/A)

	Not at all helpful	Slightly helpful	Moderately helpful	Very helpful	N/A
General practitioner					
Specialist doctor or specialist nurse					
Printed leaflet or booklet (from a healthcare professional)					
Research articles or books					
Patient/parent organisation					
Support group or forum					
Online blogs or social media (e.g. Facebook)					
Internet search (e.g. via Google)					

(4) Trustworthiness of information

1. Please rate how trustworthy you have found information accessed or received from the following sources about your child's condition. (If you have not accessed or received information from a listed source, please select N/A)

Information source	Not at all trustworthy	Slightly trustworthy	Moderately trustworthy	Very trustworthy	N/A
General practitioner					
Specialist doctor or specialist nurse					
Printed leaflet or booklet (from a healthcare professional)					
Research articles or books					
Patient/parent organisation					
Support group or forum					
Online blogs or social media (e.g. Facebook)					
Internet search (e.g. via Google)					

(5) Satisfaction with information

1. Overall, are you satisfied with the information you have received about your child's condition?

a. Not at all
b. A little
c. Quite a bit
d. Very much

(6) Information topics

4. Would you like more information about any of the following topics? (Please select up to five topics)

a. Treatment options
b. Specialist medical centres
c. Physical development
d. Intellectual development
e. Diet and feeding
f. Exercise
g. Sleep
h. Quality of life
i. Positive information about child's full potential
j. Support with school or education
k. Financial support
l. Patient organisations/support groups
m. How to meet other parents or families
n. None of the above

(C) STROBE CHECKLIST

STROBE Statement—Checklist of items that should be included in reports of ***cross-sectional studies***

	Item No	Recommendation	Page No
**Title and abstract**	1	(*a*) Indicate the study's design with a commonly used term in the title or the abstract	1, 3
		(*b*) Provide in the abstract an informative and balanced summary of what was done and what was found	3
**Introduction**			
Background/rationale	2	Explain the scientific background and rationale for the investigation being reported	5-6
Objectives	3	State specific objectives, including any prespecified hypotheses	6
**Methods**			
Study design	4	Present key elements of study design early in the paper	6-7
Setting	5	Describe the setting, locations, and relevant dates, including periods of recruitment, exposure, follow-up, and data collection	6-7
Participants	6	(*a*) Give the eligibility criteria, and the sources and methods of selection of participants	8
Variables	7	Clearly define all outcomes, exposures, predictors, potential confounders, and effect modifiers. Give diagnostic criteria, if applicable	7-8
Data sources/ measurement	8*	For each variable of interest, give sources of data and details of methods of assessment (measurement). Describe comparability of assessment methods if there is more than one group	7-8
Bias	9	Describe any efforts to address potential sources of bias	n/a
Study size	10	Explain how the study size was arrived at	9-10
Quantitative variables	11	Explain how quantitative variables were handled in the analyses. If applicable, describe which groupings were chosen and why	10
Statistical methods	12	(*a*) Describe all statistical methods, including those used to control for confounding	9-10
		(*b*) Describe any methods used to examine subgroups and interactions	9-10
		(*c*) Explain how missing data were addressed	9-10
		(*d*) If applicable, describe analytical methods taking account of sampling strategy	n/a
		(*e*) Describe any sensitivity analyses	n/a

**Results**			
Participants	13*	(a) Report numbers of individuals at each stage of study—eg numbers potentially eligible, examined for eligibility, confirmed eligible, included in the study, completing follow-up, and analysed	10-12
		(b) Give reasons for non-participation at each stage	n/a
		(c) Consider use of a flow diagram	n/a
Descriptive data	14*	(a) Give characteristics of study participants (eg demographic, clinical, social) and information on exposures and potential confounders	6, 10-11
		(b) Indicate number of participants with missing data for each variable of interest	11 (and Tables 3, 4)
Outcome data	15*	Report numbers of outcome events or summary measures	n/a
Main results	16	(*a*) Give unadjusted estimates and, if applicable, confounder-adjusted estimates and their precision (eg, 95% confidence interval). Make clear which confounders were adjusted for and why they were included	14-17 (Tables 3, 4)
		(*b*) Report category boundaries when continuous variables were categorized	n/a
		(*c*) If relevant, consider translating estimates of relative risk into absolute risk for a meaningful time period	n/a
Other analyses	17	Report other analyses done—eg analyses of subgroups and interactions, and sensitivity analyses	n/a
**Discussion**			
Key results	18	Summarise key results with reference to study objectives	20-21
Limitations	19	Discuss limitations of the study, taking into account sources of potential bias or imprecision. Discuss both direction and magnitude of any potential bias	22-23
Interpretation	20	Give a cautious overall interpretation of results considering objectives, limitations, multiplicity of analyses, results from similar studies, and other relevant evidence	21-22
Generalisability	21	Discuss the generalisability (external validity) of the study results	22-23
**Other information**			
Funding	22	Give the source of funding and the role of the funders for the present study and, if applicable, for the original study on which the present article is based	25

*Give information separately for exposed and unexposed groups. **Note:** An Explanation and Elaboration article discusses each checklist item and gives methodological background and published examples of transparent reporting. The STROBE checklist is best used in conjunction with this article (freely available on the Web sites of PLoS Medicine at http://www.plosmedicine.org/, Annals of Internal Medicine at http://www.annals.org/, and Epidemiology at http://www.epidem.com/). Information on the STROBE Initiative is available at www.strobe-statement.org.

(D) ORGANISATIONS SUPPORTING RECRUITMENT

The following organisations supported the recruitment of participants across Europe:

Spina Foundation (Poland), Borys the Hero Foundation (Poland), Fundacja TAK dla Samodzielności (Poland), Uniwersytet Medyczny im. Piastów Śląskich we Wrocławiu (Poland), Collegium Medicum Uniwersytetu Mikołaja Kopernika (Poland), The Cleft Lip and Palate Association (UK), The Children's Heart Federation (UK), Children's Heartbeat Trust (UK), Down's Syndrome Association (UK), Down Syndrome International (UK), Fondazione Toscana Gabriele Monasterio (Italy), Associazione "Un cuore, un mondo" (Italy), Associazione "Trisomia 21 Onlus" (Italy), Azienda Ospedaliero Universitaria Pisana (Italy), Arbeitsgemeinschaft Spina Bifida und Hydrocephalus (Germany), University Hospital Magdeburg (Germany), Hjerteforeningens børneklub (Denmark), Rygmarvsbrokforeningen (Denmark), Downs syndrom Danmark (Denmark), Landsforeningen Læbe- Ganespalte (Denmark), Pais21 (Portugal), Associação Spina Bifida e Hidrocefalia de Portugal (Portugal), Associação Coração Feliz (Portugal), Associação Portuguesa dos Amigos das Crianças Portadoras de Fendas Lábio-Palatinas (Portugal), The Foundation for the Promotion of Health and Biomedical Research of Valencia Region (FISABIO, Spain), Universitair Ziekenhuis Antwerpen (Netherlands), Vereniging voor Aangeboren Gelaatsafwijkingen (Netherlands), Het Centrum voor Ontwikkelingsstoornissen (Netherlands/Belgium), Spina Bifida Hydrocephalus Belgium, International Federation for Spina Bifida and Hydrocephalus (Belgium), Vereniging voor Aangeboren Gelaatsafwijkingen (Belgium), Universitair Ziekenhuis Antwerpen (Belgium), Hrvatski savez za rijetke bolesti (Croatia), Veliko srce malom srcu (Croatia), Hrvatska zajednica za Down sindrom (Croatia), Udruga roditelja djece s rascjepom usne i/ili nepca OSMIJEH (Croatia), Udruga Aurora- Udruga roditelja i djece sa spinom bifidom (Croatia), Patientenvereniging Aangeboren Hartaandoeningen (Croatia), De 'Stichting Downsyndroom' (Croatia).
